# Investigation on the Compressive Behavior of Hybrid Polyurethane(PU)-Foam-Filled Hyperbolic Chiral Lattice Metamaterial

**DOI:** 10.3390/polym15092030

**Published:** 2023-04-25

**Authors:** Qingguo He, Yuliang Hou, Xiaomeng Li, Shuang Li, Liang Meng

**Affiliations:** 1 School of Mechanical and Power Engineering, Zhengzhou University, Science Road 100, Zhengzhou 450001, China; 2 School of Mechanics and Safety Engineering, Zhengzhou University, Science Road 100, Zhengzhou 450001, China; 3 State IJR Center of Aerospace Design and Additive Manufacturing, School of Mechanical Engineering, Northwestern Polytechnical University, Xi’an 710072, China

**Keywords:** hybrid metamaterial, hyperbolic chiral lattice, polyurethane (PU) foam, additive manufacturing, freeze casting

## Abstract

In this study, a novel hybrid metamaterial has been developed via fulfilling hyperbolic chiral lattice with polyurethane (PU) foam. Initially, both the hyperbolic and typical body-centered cubic (BCC) lattices are fabricated by 3D printing technique. These lattices are infiltrated in a thermoplastic polyurethane (TPU) solution dissolved in 1,4-Dioxane, and then freeze casting technique is applied to achieve the PU-foam-filling. Intermediate (IM) layers possessing irregular pores, are formed neighboring to the lattice-foam interface. While, the foam far from the lattice exhibits a multi-layered structure. The mechanical behavior of the hybrid lattice metamaterials has been investigated by monotonic and cyclic compressive tests. The experimental monotonic tests indicate that, the filling foam is able to soften the BCC lattice but to stiffen the hyperbolic one, further to raise the stress plateau and to accelerate the densification for both lattices. The foam hybridization also benefits the hyperbolic lattice to prohibit the property degradation under the cyclic compression. Furthermore, the failure modes of the hybrid hyperbolic lattice are identified as the interface splitting and foam collapse via microscopic analysis. Finally, a parametric study has been performed to reveal the effects of different parameters on the compressive properties of the hybrid hyperbolic lattice metamaterial.

## 1. Introduction

Lightweight lattice metamaterials are an emerging class of architected materials that open up new opportunities to achieve tailored mechanical properties with minimal weight and cost [1,2]. Generally, the lattice metamaterials are periodic aggregates of artificially-designed unit cells, offering high specific stiffness and strength, excellent energy absorption and impact resistance, multi-functionality etc. Furthermore, recent progress in additive manufacturing (AM) techniques facilitates the design and fabrication of complex lattice metamaterials [3,4]. Among these designs, chiral lattice metamaterials [5,6] have attracted rising interest for their unique “chirality”, introducing tension-twist or compression-twist coupling features. Several novel designs of chiral lattice metamaterials have been successfully applied to engineering structures, e.g., lightweight energy absorber [7], tailored vibration damper [8], etc.

**Figure 1:**
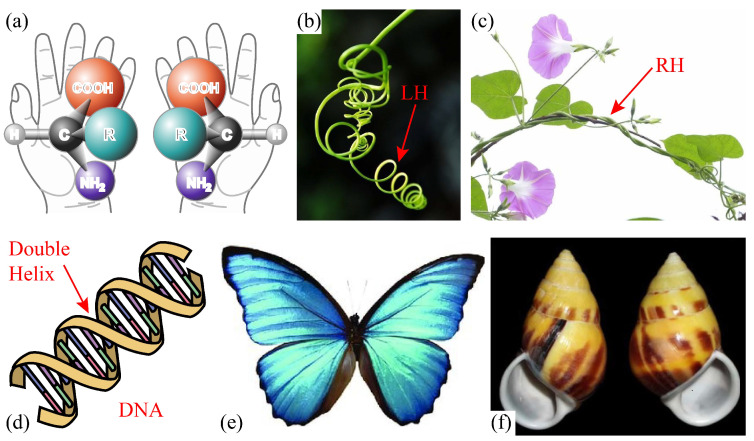
Chirality in nature: (**a**) chiral enantiomers of a generic amino acid [16] , (**b**) left-handed (LH) vine of Luffa aegyptica, (**c**) right-handed (RH) vine of Morning glory, (**d**) double helix of DNA [17], (**e**) Morpho rhetenor butterfly wings [18] and (**f**) left- and right-handed shells of Amphidromus perversus [19].

Chirality is originally observed in natural non-centrosymmetric materials and structures as shown in Figure 1. These natural chiralities inspire scientists and engineers to propose novel structural designs [9]. Initially, a set of two-dimensional (2D) chiral lattices have been developed to obtain negative Poisson’s ratio [10,11,12], enhanced fracture toughness [13] and outstanding energy absorption. Besides that, other 2D chiral lattices including tetra-chiral and tri-chiral lattices, as well as their anti-chiral lattices [14,15] have been proposed to favor structures with unique mechanical features.

Recently, several three-dimensional (3D) chiral lattices have been designed to obtain special mechanical behaviors. Furthermore, advanced AM techniques open up a possibility of manufacturing these lattices, and further boost the design of 3D chiral lattices with multi-functionality. Thereinto, a set of 3D chiral lattices, such as tetra-chiral block [5], tetra-chiral cylindrical [20], cubic chiral [21,22], hyperbolic chiral [6], have been proposed to achieve tension-twist or compression-twist coupling. The compressive behavior of a typical chiral lattice metamaterial is usually classified into three stages: (i) a linear elastic stage resulted from the twisting and bending of the cell rods or frames, (ii) a nonlinear stress plateau stage referred to the recoverable buckling or twisting of the lattice cells, and (iii) a densification stage where the cell rods or frames begin to break, and the already-buckled cells rapidly compress against each other to nearly full density, consequently leading to a steep rising stress. Compared with other types of lattices, all deformed rods within these 3D chiral lattices rotate with the same angle, when subjected to compressive loads in given directions [23]. Moreover, the lattice cells are able to fully recover their shapes when the compressive loads are removed prior to the densification stage. Their underlying deformation mechanisms have been investigated via numerical and experimental approaches [6,22]. In addition, these special features make these chiral lattices to generate a large and stable plateau stress when they are compressed or impacted with low velocity. Therefore, these tension-twist and compression-twist chiral lattices are believed to be promising materials in energy absorption.

Similarly, lightweight and hyperelastic metallic and polymeric foams are used to dissipate impact energy due to their superior energy absorption capacity [24,25]. Since their stiffness and hardness are relatively smaller, they commonly possess a lower but longer stress plateau stage than the lattice materials. However, the buckling of the foam cells appearing within the stress plateau stage, is frequently irrecoverable, except visco-elastic foams. Although these features enable foams to own higher energy-absorption efficiency (namely, specific energy absorption, SEA), they cannot recover after plastic crushing and provide full protection during the subsequent impact events. This single-impact constraint limits the usage of foams in engineering structures.

An emerging class of hybrid materials that overcomes this limitation, has been developed by combining lattices and closed-cell foams [26,27,28,29,30,31,32,33]. A pyramidal lattice core that uses polyurethane (PU) foam as filler, was developed to improve the energy absorption capacity and impact resistance [26]. Quasi-static compressive and dynamic low-velocity impact (LVI) tests had been performed on the foam-filled and unfilled lattices. The experimental results indicated that, the foam-filled one possesses a better load-carrying capacity, compared to the pure lattice and PU foam. Although the filling foam certainly enhanced the stiffness of the pyramidal lattice, it had no obvious improvement on the impact resistance and energy absorption according to the LVI tests. Tetrahedral truss lattices were filled by ceramic-particle-reinforced-PU (PU/C) foams to obtain composite lattice sandwich cores [29]. Quasi-static and dynamic compressive responses were studied to analyze the strain rate and ceramic particle reinforcement effects. The foam-filled lattice with higher weight fraction of ceramic particle was found to exhibit the highest energy absorption capacity under the quasi-static compression. The increasing ceramic particles enable to reduce the transmitted impulse, while absorb no additional energy under the dynamic compression. Moreover, obvious recovery occurred on the foam-filled lattice during the unloading stage of the dynamic compression. Similarly, a compliant foam was infiltrated inside two different auxetic lattices (namely, Hexaround and cubic anti-chiral lattices), to manufacture hybrid lattices [30]. Their mechanical properties were investigated via experimental and numerical approaches. The filling foam was found to increase the stiffness, strength and energy adsorption efficiency of the auxetic lattices, and further to modify the failure mode from layerwise crushing to shear band breaking during the compression process.

Inspired by the aforementioned researches, the current study aims to develop a novel hybrid PU-foam-filled hyperbolic chiral lattice metamaterial to improve the load-carrying and energy-absorption performance. Two classes of lattice cells, i.e., body-centered cubic (BCC) truss lattice and hyperbolic chiral lattice, are designed and manufactured using AM technique. Subsequently, the PU foam with a more uniform and finer porous structure is obtained by freeze casting technique, and meanwhile filled into the pre-fabricated lattices. Monotonic and cyclic compressive tests are carried out on the foam-filled lattices, as well as the pure lattices and foam, to investigate their compressive behavior and failure mechanism. Finally, a parametric study is performed to analyze the effects of the structural parameters associated with the hyperbolic lattice and the PU foam density on the mechanical behavior of the hybrid lattice metamaterial.

## 2. Materials and Methods

### 2.1. Design and Additive Manufacturing of BCC and Hyperbolic Lattices

**Figure 2:**
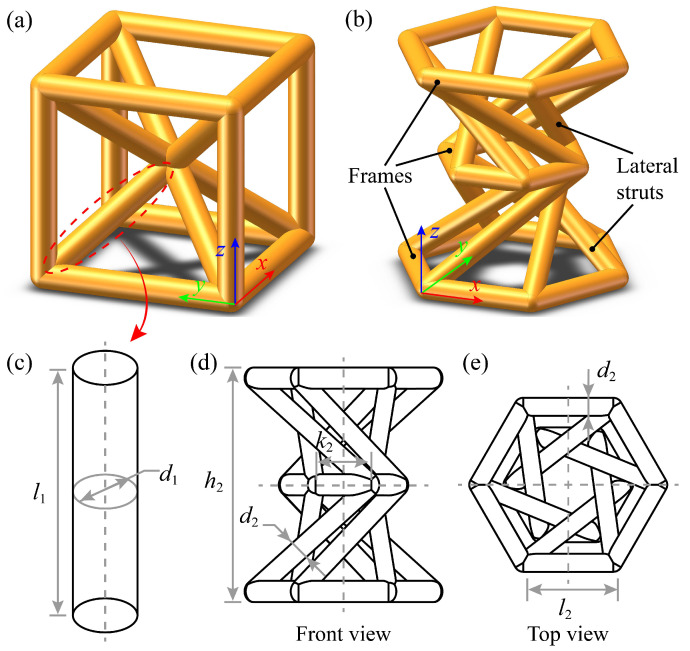
Illustration of: (**a**) BCC lattice cell and (**b**) hyperbolic lattice cell (topology parameters: n=6, inc.=±2). The parametric definitions of these cells are provided in (**c**), (**d**) and (**e**), respectively.

In this study, two types of lattice unit cells: a typical heiral body-centered cubic (BCC) truss lattice and a chiral hyperbolic lattice, are designed and manufactured, as shown in Figure 2. For each lattice, several design parameters are selected to describe the strut of each unit cell. To be specific, 2 parameters, including the truss diameter $d_{1}$ and length $l_{1}$, are adopted to describe the BCC cell, as shown in Figure 2c. While, the topology of the hyperbolic lattice cell is described by two parameters, *n* and inc. [6]. Thereinto, *n* refers to the number of edges within the polygonal scaffolds that are located at the top, middle and bottom frames of the hyperbolic lattice cell. Another parameter inc. denotes the increment in the serial numbers of two neighboring scaffolds, and lateral struts are created to connect these scaffolds. The value of inc. is assumed to be positive if the connection is counter-clockwise, and negative otherwise. Detailed information is provided in our previous study [6].

In Figure 2b, a hyperbolic lattice cell of n=6 and inc.=±2 is generated accordingly. In addition, 4 other parameters are used to describe the dimension of the frames and lateral struts, as illustrated in Figure 2d,e. $d_{2}$ refers to the truss diameter of the frames and lateral struts, $h_{2}$ is the height between the top and bottom frames. $l_{2}$ and $k_{2}$ denote the side lengths of the regular polygons corresponding to the top (bottom) and middle frames, respectively.

**Figure 3:**
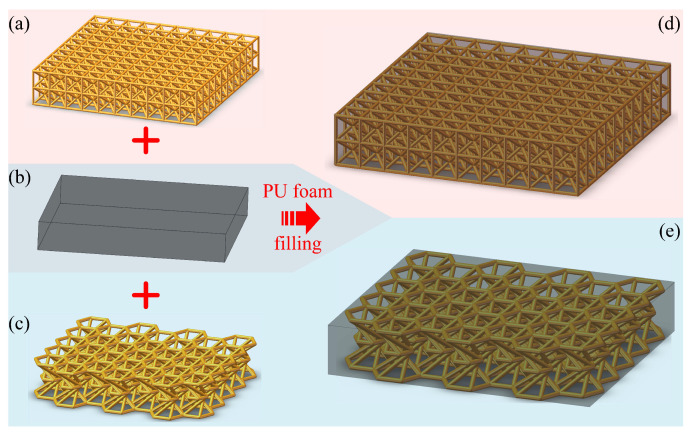
Illustration of: (**a**) BCC lattice, (**b**) PU foam and (**c**) hyperbolic lattice, as well as the strategy to obtain the hybrid PU-foam-filled (**d**) BCC and (**e**) hyperbolic lattice metamaterials.

As shown in Figure 3a,c, 10×10×2 BCC cells and 7×5 hyperbolic cells are used to array into the BCC and hyperbolic lattices, respectively. To ensure the same volume of these two lattices, the dimensions are set as 52 × 52 × 11 mm and 56 × 48.63 × 11 mm for the BCC and hyperbolic ones, respectively. Hence, the total material volume of each lattice is approximately 3910 mm^3^. Once the lattices are manufactured, the counterpart within the hybrid metamaterials is obtained by filling PU foam, as illustrated in Figure 3d,e.

Samples of both lattices have been fabricated using a 3D printer SPS600B (produced by Shaanxi Hengtong Intelligent Machine Co., Ltd., Xi’an, China) with Stereo Lithography Appearance (SLA) technique. This 3D printer possesses the maximum resolutions of 0.05 mm and 0.08 mm for planar and vertical directions, respectively. The photosensitive resin C-UV 9400A (provided by Dongguan Aide Polymer Material Technology Co., Ltd., Dongguan, China) is used to manufacture these lattices, the density is 1.11 g/cm^3^ and the viscosity is 275 cps at room temperature. As a type of SLA 3D priting ink, the photosensitive resin can be cured by the exposure of UV light with a wavelength of 355 nm during the printing process. The speed, power and size of the printing spot are set to 6000 mm/s, 0.5 w and 0.12 mm, respectively. Moreover, the density of post-cured resin is 1.12 g/cm^3^, the Young’s modulus is 2649 MPa, and the Possion’s ratio is 0.4. After the additive manufacturing, the printed lattices are placed into a ethanol bath to remove the uncured resin. And then, these lattices are dried and exposed to UV light for 30 min, to guarantee their stable performance.

### 2.2. Fabrication of Hybrid PU-Foam-Filled Lattice Metamaterials

**Figure 4:**
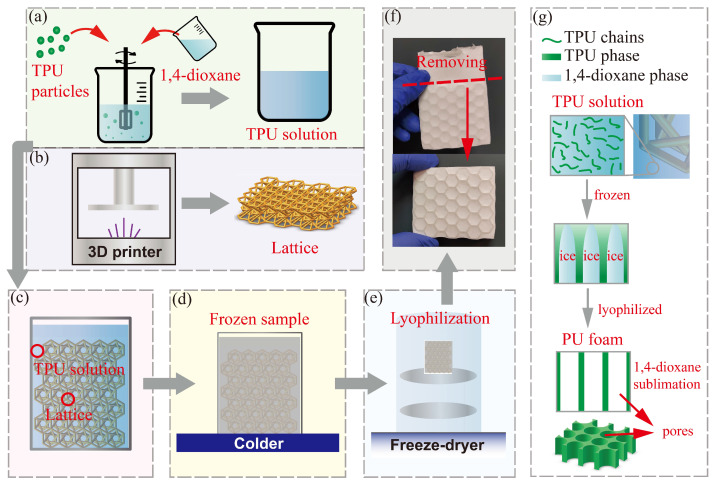
Schematic illustration of the freeze casting process to achieve the PU-foam-filling within the printed lattice: (**a**) the TPU/1,4-dioxane solution, (**b**) the 3D-printed lattice, (**c**) the hyperbolic lattice filled into TPU solution, (**d**) the frozen sample, (**e**) the lyophilization process, (**f**) the obtained hybrid PU-foam-filled hyperbolic lattice and (**g**) the mechanism of the PU-foam-filling.

In the study, PU foam is filled into the 3D-printed lattices via freeze casting technique [34,35], as illustrated in Figure 4. Thermoplastic polyurethane (TPU) elastomers Estane 58887 (produced by Lubrizol Advanced Materials, Inc., Cleveland, OH, USA) are initially mixed into a 1,4-dioxane solution (produced by Tianjin Kemiou Chemical Reagent Co., Ltd., Tianjin, China), with a TPU concentration of 10 *w*/*v*% (namely $c_{PU}=10\%$). The mixture is placed into a magnetic stirrer, and then is stirred thoroughly (temperature: 60 °C, speed: 600 r/min). After 8 h of stirring, the TPU particles have been completely dissolved into the 1,4-dioxane solution, as shown in Figure 4a.

Prior to the PU-foam-filling process, the printed lattice(see Figure 4b) is placed into a rectangular mold with the internal dimension of 12×58×80 mm. The TPU solution is poured into the mold, and the solution level is required to exceed the whole lattice structure (see Figure 4c). Subsequently, the fulfilled mold is frozen at −80 °C for 72 h to form an 1,4-dioxane ice template (see Figure 4d), followed by lyophilization at −45 °C for 72 h by a freeze-dryer FreeZone 6L (produced by Labconco Co., Kansas City, MO, USA) to sublimate the 1,4-dioxane phase, as illustrated in Figure 4e. Finally, the hybrid PU-foam-filled hyperbolic lattice (see Figure 4f) is obtained after demolding and removing the redundant foam. The mechanism of freeze casting process to achieve the PU-foam-filling is illustrated in Figure 4g. The TPU chains dissolved in 1,4-dioxane gather to form TPU phase, since the chains are extruded and assembled by the growing 1,4-dioxane ice crystals during the freezing process. After the lyophilization, the 1,4-dioxane ice frame sublimates and the TPU phase remains, obtaining the PU foam with porous microstructure like balsa wood. Obviously, pores within the resulting PU foam is a replica of 1,4-dioxane ice crystals, that is, their morphology mainly depends on the 1,4-dioxane ice crystals growing process.

**Figure 5:**
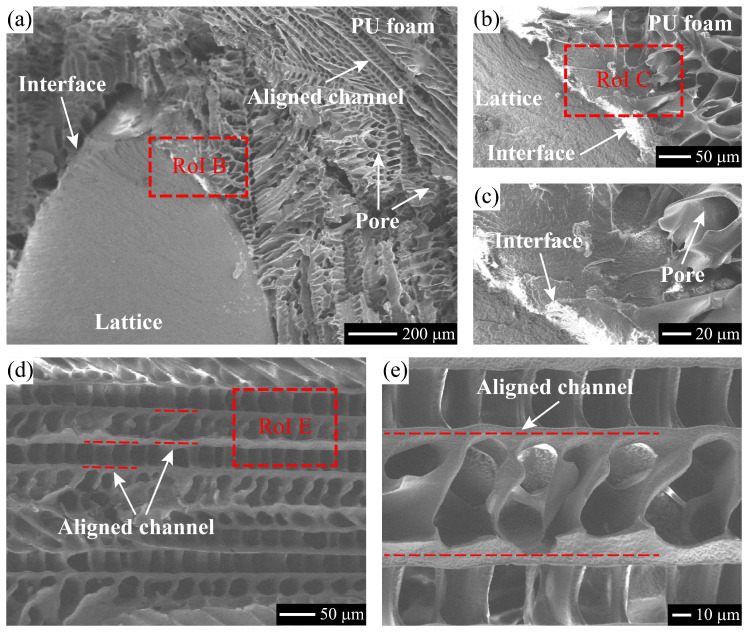
SEM graphs of: (**a**–**c**) the hybrid PU-foam-filled hyperbolic lattice and (**d**,**e**) the pure PU foam ($c_{PU}$ = 10%). (**b**), (**c**) and (**e**) are taken from RoIs B, C and E in (**a**), (**b**) and (**d**), respectively.

Microstructures of the hybrid PU-foam-filled lattice have been analyzed by a scanning electron microscope (SEM) Zeiss-sigma 300, as shown in Figure 5a–c. The dense and porous areas observed in Figure 5a, represent the lattice and PU foam, respectively. Especially, the PU foam not adjacent to the lattice, mostly form a multi-layered structure. Within the multi-layered structure, several aligned channels appear due to the freezing process of the TPU solution [36]. Moreover, an obvious interface marked by regions of interest (RoIs) B and C in Figure 5a,b, appears between the lattice and PU foam. Several intermediate (IM) layers are found in the PU foam near to the interface, where irregular pores are distributed in a non-uniform manner, as illustrated in Figure 5b,c.

In addition, the pure PU foam with $c_{PU}$ = 10% has been fabricated, and the microstructure has been characterized by SEM, as shown in Figure 5d,e. The multi-layered structure including aligned channels and pores, has also been observed in the pure PU foam. Similar phenomena have been reported by [36,37], and bio-inspired multi-functional cellure materials have been developed accordingly. Furthermore, the ice crystal growing process is tunable and mostly determined by the temperature gradient during the freezing process. Layered cellular foams or plastics with orthogonal channels, or more complex porous structures can be fabricated by the directional freeze casting technique. And, the excellent mechanical properties, such as energy absorption, impact resistance, damping etc., have been verified by literatures [38,39,40,41].

## 3. Experimental Procedure

### 3.1. Specimen Preparation

In this study, experimental compressive tests have been performed to characterized the mechanical behavior of BCC and hyperbolic lattices, PU foams and hybrid PU-foam-filled lattices. In order to analyze the design parameters (mainly structural parameters of the hyperbolic lattice and PU foam density) on the compressive properties, several groups of specimens are included in the whole testing plan, as listed in Table 1.

**Figure 6:**
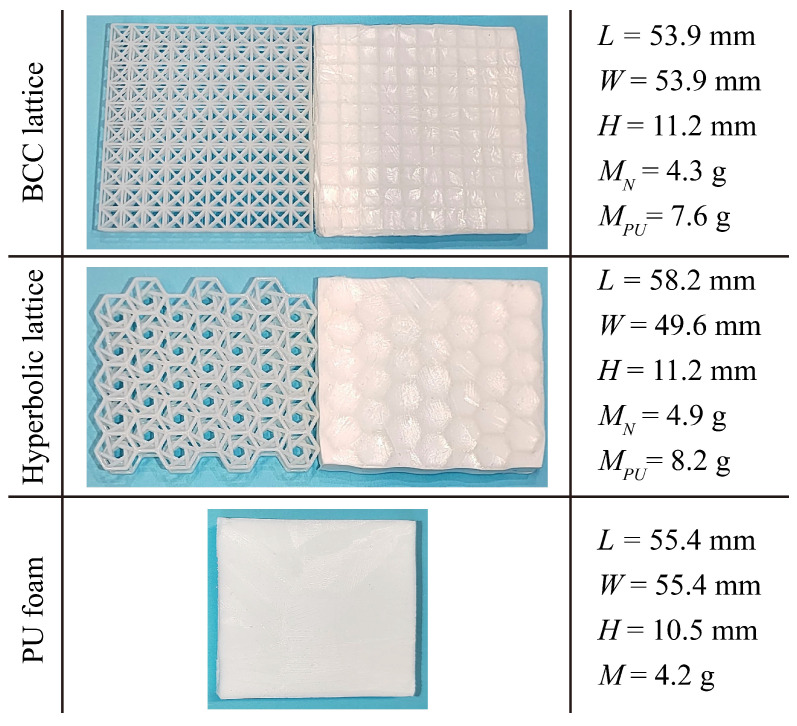
Illustration and physical information of the compressive specimens manufactured by the hybrid PU-foam-filled BCC (specimen group: BCC-PU-1) and hyperbolic (specimen group: Hyperbolic-PU-1) lattices, as well as the pure PU foam (specimen group: PU-2).

Cuboid specimens have been prepared for the compressive tests, and their geometrical and mass details are given in Figure 6. *L*, *W* and *H* are the length, width and height of the cuboid specimens, respectively. $M_{N}$ refers to the average mass of the BCC or hyperbolic lattices, $M_{PU}$ denotes that of the PU-foam-filled ones. The compressive specimens of BCC and hyperbolic lattices are fabricated via 3D printing, and the ones made of PU foam are directly prepared by freeze casting technique. The specimens of hybrid PU-foam-filled lattices are obtained by the method presented in Section 2.2.

### 3.2. Compressive Testing

**Figure 7:**
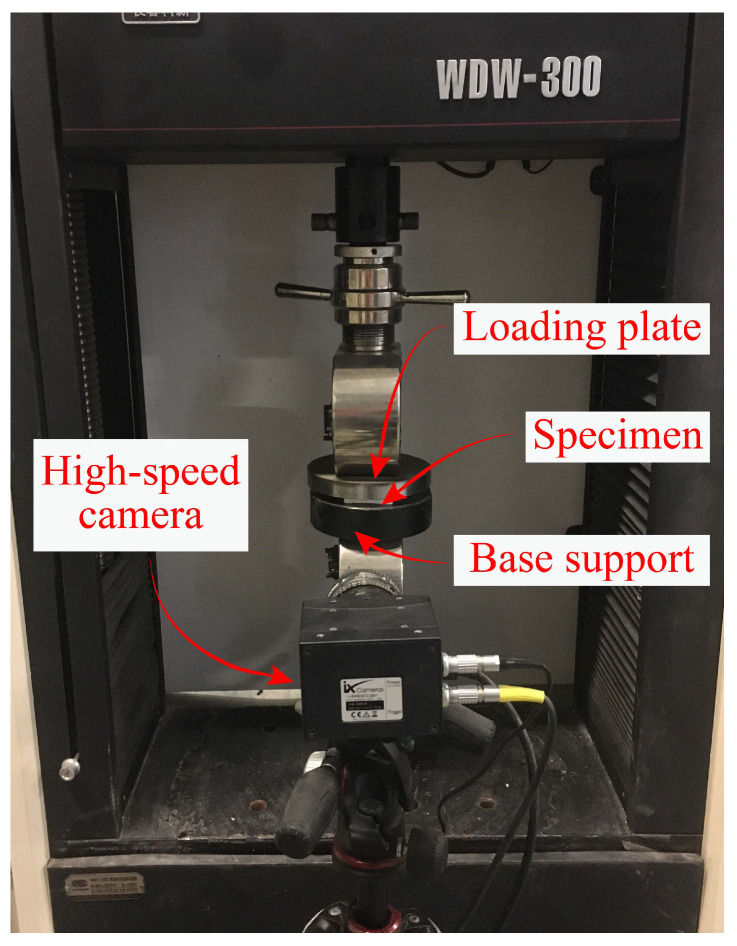
Experimental testing platform used to perform the compressive tests.

Monotonic compressive tests have been performed on the obtained specimens by a universal testing machine WDW-300, as illustrated in Figure 7. During the compression process, the specimen is freely placed on the base support, and a compressive displacement is prescribed on the loading plate with a displacement rate of 0.5 mm/min at room temperature. Displacement-force curves are directly obtained from the universal testing machine. Meanwhile, a high speed camera I-speed 221 has been employed to record the compression process.

Additionally, cyclic compressive tests have been carried out by the aforementioned testing platform, to investigate the resistance to property-degradation under the cyclic loads. The cyclic tests includes 15 single loading and unloading processes. During each loading process, the compressive displacement is defined with a certain value that ensures the specimen to exceed the linear elastic stage and further to enter the nonlinear stress plateau stage. And, the loading rate remains the same with the monotonic tests. While, the rate increases to 5 mm/min during the unloading process, to speed up the cyclic tests. Cyclic compressive responses are recorded for the specimens made by lattices and hybrid PU-foam-filled lattices, and are discussed in Section 4.

## 4. Results and Discussion

### 4.1. Monotonic Compressive Responses

**Figure 8:**
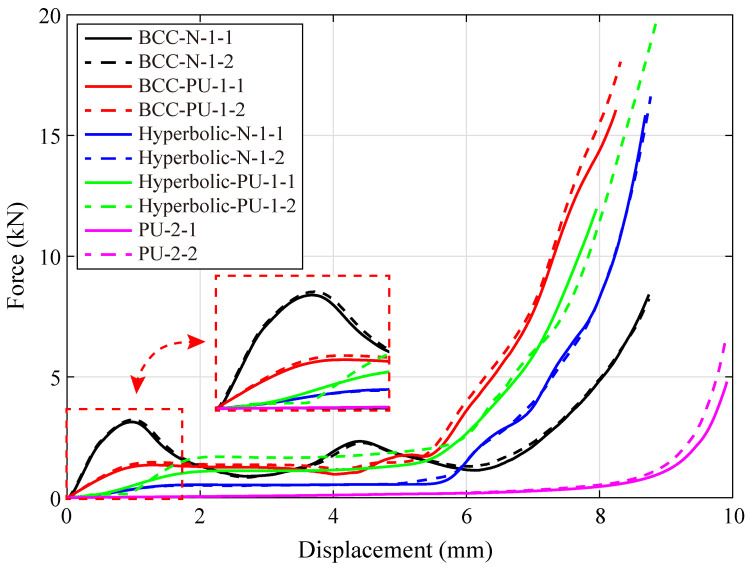
Monotonic compressive displacement-force curves of the specimens made of lattices (BCC-N-1 and Hyperbolic-N-1), hybrid PU-foam-filled lattices (BCC-PU-1 and Hyperbolic-PU-1) and PU foams (PU-2).

Figure 8 shows the monotonic compressive responses of the specimens fabricated by lattices, hybrid PU-foam-filled lattices and PU foams. The aforementioned three compression stages clearly appear within these displacement-force curves, except that of the PU foam specimens. While, the curve of the PU foam enters the stress plateau stage after an extremely short elasticity. Moreover, the PU foam begins the rapid densification when the compressive displacement reaches to about 9 mm. It is mainly due to that, the cell wall within the PU foam possesses a quite small thickness, resulting in relatively low bending and buckling strengths. Hence, the recoverable buckling quickly occurs into the foam cells, where the air is squeezed out accordingly. Subsequently, the cell walls deform fiercely, further twist and collapse.

**Figure 9:**
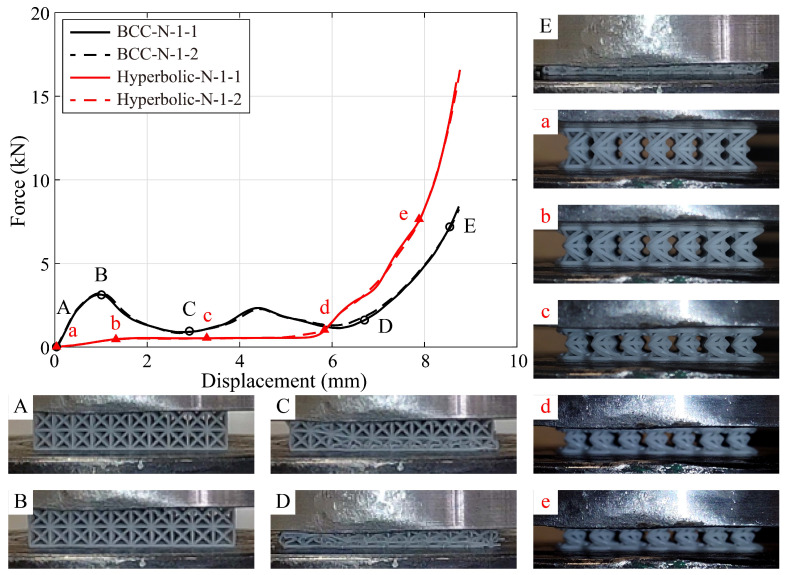
Monotonic compressive responses and failure processes of specimens BCC-N-1 and Hyperbolic-N-1. Snapshots A–E and a–e illustrate the tested specimens BCC-N-1 and Hyperbolic-N-1 under different compressive displacements, corresponding to the black circles and red triangles on the displacement-force curves.

**Figure 10:**
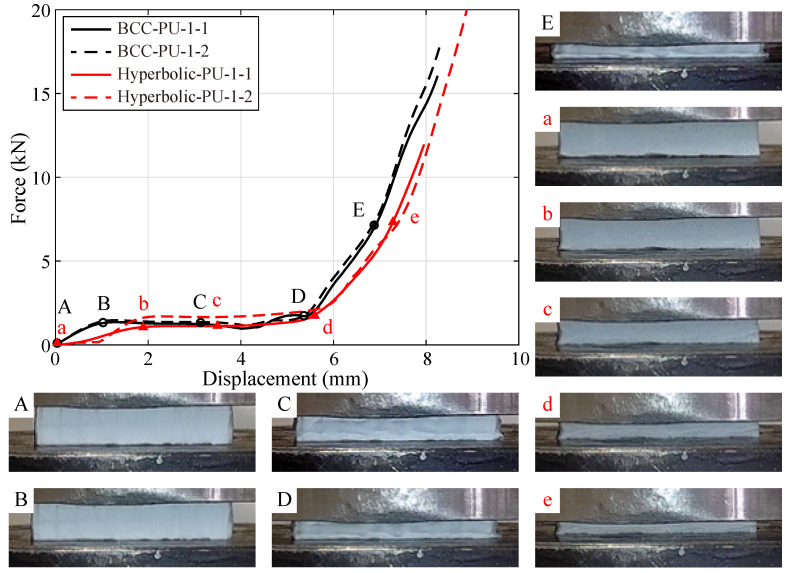
Monotonic compressive responses and failure processes of specimens BCC-PU-1 and Hyperbolic-PU-1. Snapshots A–E and a–e illustrate the tested specimens BCC-PU-1 and Hyperbolic-PU-1 under different compressive displacements, corresponding to the black circles and red triangles on the displacement-force curves.

As illustrated in Figure 8, both the primitive and hybrid hyperbolic lattices possess a longer elastic stage than the BCC ones. Moreover, the specimens fabricated by BCC lattice (BCC-N-1) are found to be stiffer than those of hybrid ones (BCC-PU-1), prior to the linear elastic limit. This may be due to the BCC lattice being covered with PU foam. While, the specimens of hyperbolic lattice (Hyperbolic-N-1) possess a lower stiffness than those filled with PU foam (Hyperbolic-PU-1). These results indicate that, the PU foam hybridization softens the BCC lattice, but stiffens the hyperbolic one. The possible explanation of this discrepancy is attributed to the different deformation mechanisms of the BCC and hyperbolic lattices. Thus, the deformation processes of these lattices are extracted from the experimental observations recorded by the High-speed camera, to comprehensively explore the deformation mechanisms, as shown in Figure 9 and Figure 10.

Each specimen subsequently undergoes a stress plateau stage whose steady-state value almost remains equal to the peak force of the linear elastic stage, except the primitive BCC lattice. The compressive force imposed on the BCC lattice slowly decreases but does not converge to a steady-state value, as the curve enters the stress plateau. These peak forces are identified as 3.22 kN and 1.45 kN for the primitive and hybrid BCC lattices, 0.50 kN and 1.08 kN for the primitive and hybrid hyperbolic lattices, respectively. Finally, the displacement-force curves of both primitive and hybrid (BCC and hyperbolic) lattices rapidly arrive into the densification stage when the compressive displacement already exceeds half of the specimen height. In comparison, the PU foam hybridization obviously accelerates the hybrid lattices to enter the densification stage, especially the BCC case. However, the curve starts to rise until the compressive displacement reaches to approximately 80% of the specimen height, in the pure foam case.

Snapshots A–E and a–e in Figure 9 and Figure 10 correspond to the tested specimens under different compressive displacements. Thereinto, snapshots A-B and a-b illustrate the specimens referred to the beginning and end of the linear elastic stage. It is noted that, the elastic stage of the BCC lattice mainly relies on the compressive and bending deformation of the constituent rods and trusses, as illustrated in Figure 9B. With the compressive displacement increasing, geometric nonlinearity occurs nearly along with material nonlinearity, simultaneously activating the stress plateau stage (Figure 9C). In the hybrid BCC lattice, the PU foam expends in the in-plane directions, and it further facilitates the vertical rods to undergo the buckling behavior. On the macroscale level, this leads to the reduction of the stiffness and elastic limit, as shown in Figure 10B. Subsequently, the nonlinear deformation of the foam and lattice cells (Figure 10C) occurs and forms a relatively stable stress plateau, deviating from the primitive BCC lattice.

From the snapshot in Figure 9b, the elastic deformation of the hyperbolic lattice is primarily attributed to the bending of the lateral struts and the twisting of the middle frame, significantly deviating from the BCC lattice case. In the hybrid hyperbolic lattice, the adhesion and friction between the PU foam and the lattice struts prohibit the middle frame from twisting. And then, the majority of the compressive loads concentrate on the lateral struts, and a larger bending deformation is required to reach the similar compressive displacement, as illustrated in Figure 10b. Thus, the stiffness and elastic limit of the hyperbolic lattice are obviously enhanced by the fulfilling PU foam during the elastic stage. After the peak force, the lateral struts continue to bend, and further force the middle frame to turn around. This phenomenon is clearly observed in Figure 9c. Although the lattice cells have been filled and covered with PU foam, the similar deformation process is believed to occur in the hybrid lattice (Figure 10c).

In addition, snapshots D-E and d-e reveal that the tested specimens begin and undergo the densification. As shown in Figure 9D,E, all the BCC cells are totally deformed and densified, some rods suffer severe buckling and even completely collapse. Despite the BCC cells in the hybrid lattice cannot be directly observed in Figure 10D,E, the constituent rods and trusses are likely to deform and fail in the same manner. Ultimately, the primitive and hybrid BCC lattices are compressed into a relatively small and dense form, without any possibility to restore their shape.

On the contrary, the lattice cells in Figure 9d,e, remain the hyperbolic configuration during the whole compressive testing process. Once the compressive loads have been removed, the hyperbolic lattice is found to nearly recover the original shape, and few rods breakage occurs within the lateral struts and middle frame. This feature favors the hyperbolic lattice overwhelming advantages on the energy absorption and impact-resistance performance. Similar tendency appears within the densification stage of the hybrid lattice. Furthermore, the unloading process after the compressive test indicates that, the PU foam effectively speeds up the shape recovery.

### 4.2. Cyclic Compressive Responses

**Figure 11:**
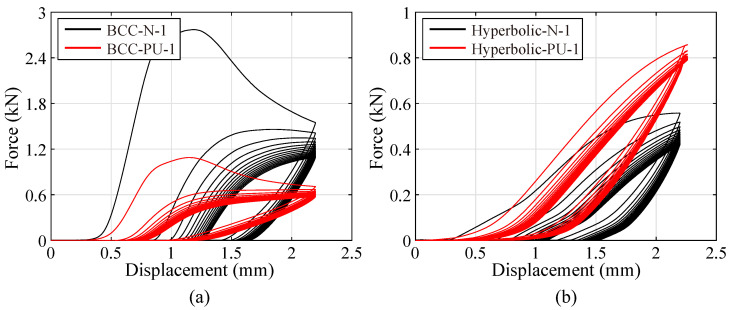
Cyclic compressive responses of: (**a**) BCC (BCC-N-1) and PU-foam-filled BCC (BCC-PU-1) lattices, (**b**) hyperbolic (Hyperbolic-N-1) and PU-foam-filled hyperbolic (Hyperbolic-PU-1) lattices.

Cyclic compressive tests have been performed on the specimens made by primitive and hybrid lattices, to comprehensively investigate the compressive behavior. During the testing process, a compressive displacement up to 20% of the specimen’s height (around 2.2 mm) has been cyclically imposed on the specimens 15 times. This displacement is able to guarantee the specimens to enter the nonlinear stage. And, the compressive loading-unloading responses are depicted in Figure 11.

During the first loading cycle, all specimens tend to behave the similar elasticity to the monotonic compression. And then, the curves decrease with different gradients during the unloading process. It is noted that, the unloading gradient possesses a positive correlation with the stiffness of each specimen. Moreover, some irreversible deformations have been produced after the first loading-unloading cycle within the specimens, especially the primitive lattices. As the loading-unloading cycles carry on, the stiffness of each specimen continuously decreases, while the unloading gradient nearly almost remains the same level. Meanwhile, the peak force of the elastic stage drops fiercely, and the irreversible deformations gradually accumulate. The curves in Figure 11a indicate that, the peak forces are reduced by 54.5% and 42.9% within the first 5 compressive cycles for the primitive and hybrid BCC lattices, respectively. And, the reduction factors are characterized as 15.1% and 6.6% for the primitive and hybrid hyperbolic lattices (Figure 11b). After that, the load-carrying capacity of each specimen decreases following a downward manner, and it finally converges to a certain value. It reveals that, either the primitive or hybrid hyperbolic lattice possesses a better resistance to the cyclic compression. Furthermore, the addition of PU foam exhibits advantages in prohibiting the property-degradation for lattice structures. This feature is certainly in favor of enriching the application of the PU-foam-filled hyperbolic lattice in the energy absorption and impact-resistance fields.

### 4.3. Failure Mechanism

**Figure 12:**
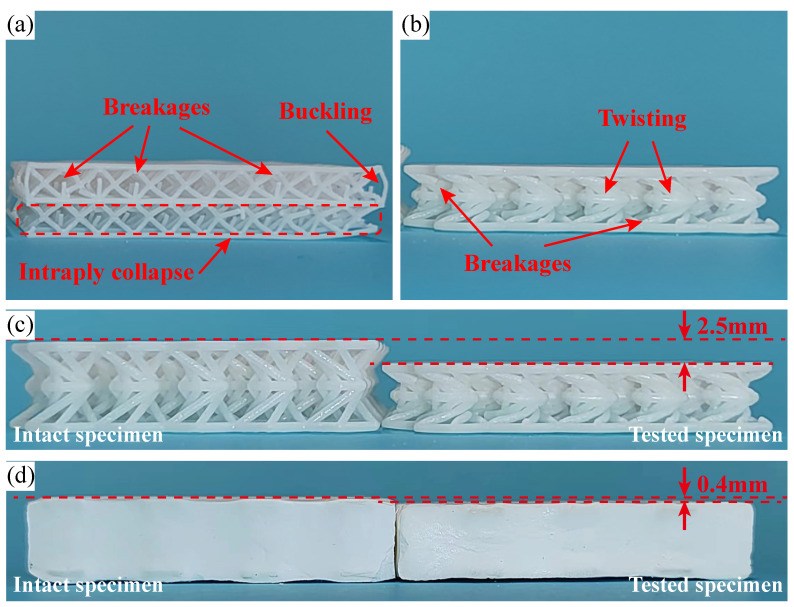
Damage morphologies of the tested specimens fabricated by: (**a**) BCC (BCC-N-1), (**b**) hyperbolic (Hyperbolic-N-1) lattices, (**c**,**d**) the intact and tested specimens fabricated by hyperbolic lattice and PU-foam-filled hyperbolic lattice.

Damage morphologies are extracted from the tested specimens by SEM analysis to systematically understand the failure mechanism. Photos have been firstly taken from the tested specimens made of BCC and hyperbolic lattices to highlight the damage location and morphology, as shown in Figure 12.

In Figure 12a, an obvious intraply collapse where all vertical rods are fully broken, has been found to occur within the bottom layer of BCC lattice. While, breakages are merely identified in a limited quantity of slanting rods. Several breakages and severe buckling appear in the vertical rods of the top layer, yet resulting in the collapse of the whole layer. These results reveal that, the failure of the BCC lattice occurs in a layerwise mode. It is also supported by Snapshots C and D in Figure 9. In comparison, the damages in the hyperbolic lattice Figure 12b are rather slight. Although each lattice cell remains in the irreversible twisting, the compressive loads only cause several rod located in the lateral struts to be broken. The photograph in Figure 12c,d presents the intact and tested specimens, and reference lines (the red dash line) are employed to mark the height of the intact specimen and tested specimen. A slight dissimilarity between the intact and tested foam filled hyperbolic lattice specimens’ heights is characterized as 0.4 mm. This is much smaller than the height variation of the primitive hyperbolic lattice (namely 2.5 mm). It indicates that, the PU foam is able to enhance the mechanical properties of the hyperbolic lattice, especially in terms of shape and dimension recovery. And, the observation on the recovery processes of different specimens also confirms that, the foam filling makes the hyperbolic lattice to possess a more efficient shape recovery rate.

**Figure 13:**
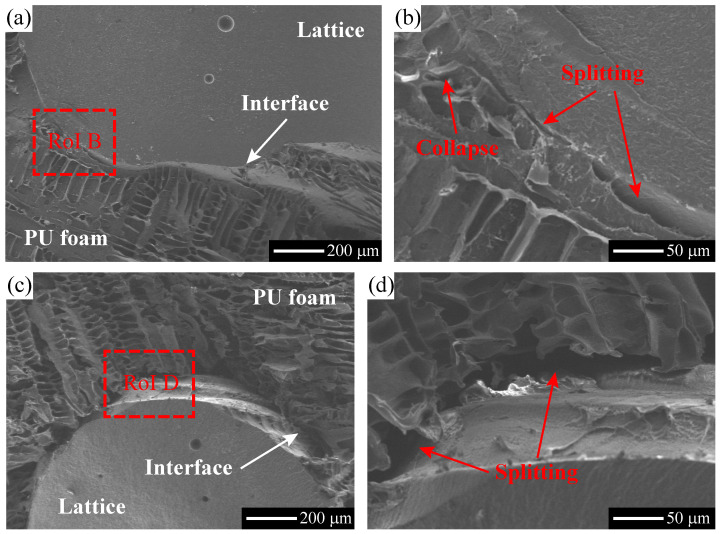
Illustration of SEM graphs taken on the tested specimen’s profiles, (**b**) and (**d**) are taken from RoIs B and D in (**a**) and (**c**), respectively.

The PU foam covers the lattice struts of the specimens made of hybrid lattices, prohibiting the damage observation. Additionally, the compressive behavior of the PU-foam-filled hyperbolic lattice is the primary interest of this study. Thus, the PU-foam-filled hyperbolic lattice has been cut, and SEM analysis are then preformed on the profile to obtain the damage morphology, as illustrated in Figure 13. Two SEM graphs have been taken on the connection areas between the PU foam and the lattice, to highlight the micro-damages and cracks, as illustrated in Figure 13a,c. No obvious damage or crack is found in the lattice rod, and the majority of foam cells still remain in good status. However, several micro-cracks or micro-voids are observed along the interface areas where two representative RoIs are selected and shown in Figure 13b,d. Within these RoIs, the PU foam has been split from the lattice rod to form the micro-cracks. Furthermore, some foam cells nearby the interface are found to be fully collapsed. Therefore, the main damage modes are identified as interface splitting and foam collapse, for the PU-foam-filled lattice subject to compressive loads.

### 4.4. Parametric Study

This section focuses on the parametric study to investigate the effects of different parameters on the compressive properties of the hybrid hyperbolic lattice metamaterials. These parameters mainly consist of the structural parameters (inc. and $d_{2}$) referred to the hyperbolic lattice and the filling PU foam density.

#### 4.4.1. Effect of Structural Parameters of Hyperbolic Lattice

Different groups of hyperbolic lattices are initially 3D-printed with varying parameters, namely inc. and $d_{2}$. Thereinto, three groups of variations have been considered for each case (inc.: 1, 1.5 and 2, $d_{2}$: 0.9, 1.0 and 1.1), and other parameters are listed in Table 1. And then, the PU foam with $c_{PU}$ = 10% is filled into these lattices by the fabrication method presented in Section 2.2. The specimen group Hyperbolic-PU-1 (inc. = 2, $d_{2}$ = 1) is considered as the reference one. Besides, the varying inc. case includes specimen groups Hyperbolic-PU-2 (inc. = 1) and Hyperbolic-PU-3 (inc. = 1.5), and the varying $d_{2}$ case corresponds to specimen groups Hyperbolic-PU-4 ($d_{2}$ = 0.9) and Hyperbolic-PU-5 ($d_{2}$ = 1.1).

**Figure 14:**
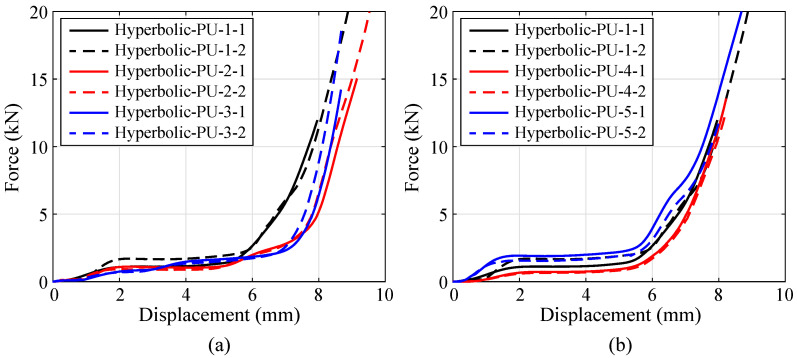
Compressive displacement-force curves obtained for the specimens made of hybrid PU-foam-filled lattices with varying parameters: (**a**) inc. and (**b**) $d_{2}$.

Figure 14 depicts the compressive displacement-force curves obtained for the aforementioned specimens. The comparison in Figure 14a indicates that, the increase of inc. almost leads to no change on the compressive responses prior to the densification stage. Subsequently, it reveals that, the hybrid lattice with higher inc. begins to be densified earlier than that with lower inc. It is due to the fact that, the hyperbolic lattice with higher inc. possesses the lateral struts whose constituent rod is at a larger angle with the vertical direction (direction z in Figure 2b). The similar compressive load causes a greater bending moment on the lateral rod in the higher inc. case. Therefore, the hybrid lattice with higher inc. is more prone to densification. In addition, the compressive curves in Figure 14b demonstrate that the hybrid lattice with larger $d_{2}$ behaves in a stiffer manner, meanwhile possesses a higher stress plateau. However, all hybrid lattices almost begin be densified at the same compressive displacement, revealing no effect of parameter $d_{2}$ on the densification stage.

#### 4.4.2. Effect of PU Foam Density

The PU foam density of the hybrid lattice mainly depends on the TPU concentration ($c_{PU}$) of the solution used for the PU-foam-filling. Hence, solutions with varying $c_{PU}$ are prepared to manufacture hybrid PU-foam-filled hyperbolic lattices, and further to analyze the effect referred to the PU foam density on the compressive properties. Similar to specimen group Hyperbolic-PU-1, two other PU foams with $c_{PU}$ = 5%, 15% are fulfilled in the same hyperbolic lattice to obtain specimen groups Hyperbolic-PU-6 and Hyperbolic-PU-7, respectively. Moreover, pure PU foams have been manufactured by the corresponding TPU solutions. These PU foams are considered as the reference group to exclude the enhancement only referred to the increasing PU foam density.

**Figure 15:**
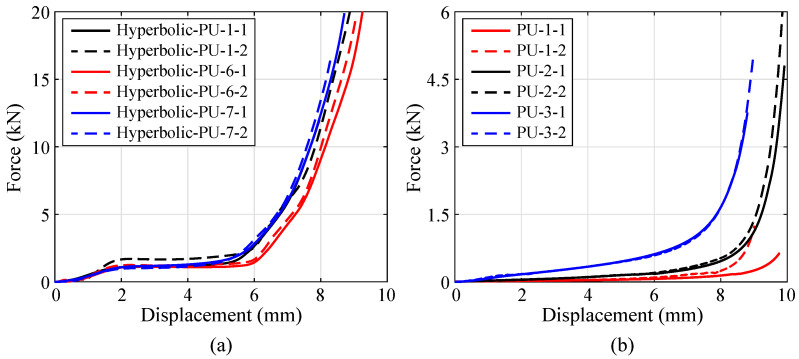
Compressive displacement-force curves obtained for the specimens made of: (**a**) hybrid PU-foam-filled lattices and (**b**) pure PU foams, with varying $c_{PU}$.

Compressive tests have been performed on these specimens, and the compressive responses are illustrated in Figure 15. As $c_{PU}$ varies from 5% to 15%, no significant difference exists within the linear elastic and stress plateau stages of the curves in Figure 15a. Moreover, the specimen with higher $c_{PU}$ enters the densification stage earlier than others. As illustrated in Figure 15b, the increase of $c_{PU}$ leads to a obvious rise of the stress plateau, as well as the acceleration of the densification, in the pure PU foam cases. Thereinto, the increasing tendency of the stress plateau differs from the hybrid lattice case. It indicates that, the linear elasticity and stress plateau mainly depends on the constituent lattice, rather than the PU foam. While, the compressive displacement to densification is quite related to the PU foam density. The higher PU foam density certainly speeds up the hybrid lattice to begin the densification.

## 5. Conclusions

In this study, a hybrid metamaterial has been developed by fulfilling PU foam into hyperbolic chiral lattice via freeze casting technique. Subsequently, the mechanical behavior was systematically investigated for the hybrid lattice metamaterial subjected monotonic and cyclic compressive loads. The damage morphologies of the tested lattice were analyzed to reveal the failure mechanism. Finally, a parametric study has been performed to explore the effects of the structural parameters referred to the hyperbolic lattice and the filling PU foam density on the compressive properties. The following conclusions can be drawn from the study:

- Hybrid lattice metamaterials have been successfully fabricated by freeze casting technique for BCC and hyperbolic lattices. An obvious interface accompanied with irregular IM layers, is formed between the lattice and the foam. The PU foam filled into the lattice possesses a multi-layered structure, including aligned channels and pores.
- The monotonic compressive curves of hybrid (BCC and hyperbolic) lattice metamaterials exhibit three typical deformation stages, same to those of the primitive lattices. The elastic stage of both lattices is prolonged by the filling PU foam, which softens the BCC lattices but stiffens the hyperbolic ones. In both cases, the PU foam hybridization rises the stress plateau, and meanwhile accelerates the lattices to enter the densification stage.
- As compression cycles performed on primitive and hybrid lattices, their load-carrying capacity decreases in a downward manner. Compared to the BCC cases, the primitive and hybrid hyperbolic lattices possess a better resistance to the cyclic compressive loads. In addition, the filling PU foam favors both BCC and hyperbolic lattices to prohibit the property-degradation during the cyclic compressive process.
- The hyperbolic lattices, especially the hybrid one, exhibit advantages on shape recovery during the unloading process. Moreover, the main failure mechanism of the hybrid hyperbolic lattice is characterized as the interface splitting and foam collapse by SEM analysis on the damage morphology.
- The parameters inc. and PU foam density ($c_{PU}$) merely affect the linear elastic and stress plateau stages of the hybrid hyperbolic lattice, while a higher inc. or $c_{PU}$ makes the lattice exhibit a smaller displacement to densification. The increasing $d_{2}$ is found to provide a complete raise on the compressive displacement-force curve, including higher elasticity and stress plateau.

## Figures and Tables

**Table 1:** Design parameters of the compressive specimens (Unit: mm).

Specimen Group	Design Parameter
BCC-N-1	$d_{1}=0.7$, $l_{1}=4.46$, $c_{PU}=0$
BCC-PU-1	$d_{1}=0.7$, $l_{1}=4.46$, $c_{PU}=10\%$
Hyperbolic-N-1	n=6, inc.=2, $d_{2}=1$, $l_{2}=5$, $k_{2}=3.1$, $h_{2}=11$, $c_{PU}=0$
Hyperbolic-PU-1	n=6, inc.=2, $d_{2}=1$, $l_{2}=5$, $k_{2}=3.1$, $h_{2}=11$, $c_{PU}=10\%$
Hyperbolic-PU-2	n=6, inc.=1, $d_{2}=1$, $l_{2}=5$, $k_{2}=3.1$, $h_{2}=11$, $c_{PU}=10\%$
Hyperbolic-PU-3	n=6, inc.=1.5, $d_{2}=1$, $l_{2}=5$, $k_{2}=3.1$, $h_{2}=11$, $c_{PU}=10\%$
Hyperbolic-PU-4	n=6, inc.=2, $d_{2}=0.9$, $l_{2}=5$, $k_{2}=3.1$, $h_{2}=10.9$, $c_{PU}=10\%$
Hyperbolic-PU-5	n=6, inc.=2, $d_{2}=1.1$, $l_{2}=5$, $k_{2}=3.1$, $h_{2}=11.1$, $c_{PU}=10\%$
Hyperbolic-PU-6	n=6, inc.=2, $d_{2}=1$, $l_{2}=5$, $k_{2}=3.1$, $h_{2}=11$, $c_{PU}=5\%$
Hyperbolic-PU-7	n=6, inc.=2, $d_{2}=1$, $l_{2}=5$, $k_{2}=3.1$, $h_{2}=11$, $c_{PU}=15\%$
PU-1	$c_{PU}=5\%$
PU-2	$c_{PU}=10\%$
PU-3	$c_{PU}=15\%$

## Data Availability

The data that support the findings of this study are available from the corresponding author upon reasonable request.
